# Systematic Review of Referral and Care Pathways for Children and Young People of Black Ethnicity Through Child and Adolescent Mental Health Services Compared With Other Ethnic Groups: An International Comparison

**DOI:** 10.1192/bjo.2024.81

**Published:** 2024-08-01

**Authors:** Babatunde Odebiyi, Cornelius Ani, Rezina Sultana, Eunice Ayodeji, Bernadka Dubicka

**Affiliations:** 1 East Lanchasire Hospitals NHS Trust, Burnley, United Kingdom; 2 Imperial College, London, United Kingdom; 3 Surrey and Borders Partnership NHS Foundation Trust, Surrey, United Kingdom; 4 Manchester University NHS Foundation Trust, Manchester, United Kingdom; 5 University of Salford, Manchester, United Kingdom; 6 Greater Manchester Mental Health Foundation Trust, Bolton, United Kingdom; 7 University of York, York, United Kingdom; 8 University of Manchester, Manchester, United Kingdom

## Abstract

**Aims:**

The review explored differences in sources of referrals and utilisation of child and adolescent mental health services (CAMHS) among children and young people (CYP) of black ethnicity compared with other ethnicities. We also explored international differences.

**Methods:**

We searched MEDLINE (through Ovid), PsycINFO, EMBASE, CINAHL, Cochrane Database of Systematic Reviews and Web of Science using a priori defined search terms to identify relevant records. We used the “Population, Exposure, and Outcome” (PEO) framework to define search terms. Pairs of authors assessed papers for inclusion, extracted the data and conducted quality assessment. The systematic review was pre-registered with PROSPERO (CRD42021249619).

**Results:**

We identified 110 studies which all had quantitative design. The results indicate that compared with other ethnic groups, CYP of black ethnicity were less likely to be screened for mental disorders, and more likely to be referred by non-voluntary sources such as social/child welfare services and juvenile justice systems. CYP of black ethnicity were also less likely to utilise all types and levels of mental health services with the exception of school-based services. CYP of black ethnicity were less likely to access psychological intervention or to be prescribed psychotropic medications. Also, CYP of black ethnicity were more likely to experience coercive treatments, and to receive poorer quality of care. These findings were similar across different countries.

**Conclusion:**

CYP of black ethnicity experience significant disadvantages across their care journeys through CAMHS. Addressing the drivers for these disadvantages is crucial for improving access to care for this group.

